# Digital universal parent training program to promote positive parenting skills – A randomized waiting-list study

**DOI:** 10.1192/j.eurpsy.2022.1465

**Published:** 2022-09-01

**Authors:** K. Mishina, M. Kinnunen, A. Heikkinen, S. Saarinen, S. Hinkka-Yli-Salomäki, A. Sinokki, T. Imberg, A. Sourander

**Affiliations:** University of Turku, Department Of Clinical Medicine, Research Center Of Child Psychiatry, University of Turku, Finland

**Keywords:** RCT, Child mental health, Digital intervention, Parent training

## Abstract

**Introduction:**

Parent training programs have high potential to promote positive parent-child relationships as well as reach and engage parents to participate. Digitally delivered programs may overcome the barriers associated with face-to-face interventions, such as stigma, logistic challenges and limited resources.

**Objectives:**

To assess the effectiveness and feasibility of digital universal parent training program for families with 3 years-old children, focusing on parenting skills and child´s behavior.

**Methods:**

A non-blinded randomized controlled trial (RCT) with two groups: (I) the intervention group, in which participants receive the parent training and (II) the waiting list group, in which participants are placed on a waiting list to receive the parent training intervention after the first follow-up measurement have been completed. Participants must meet the following inclusion criteria: a) guardians having a child age 3 years, b) participating to annual health checkup in child health clinic, c) at least one of the guardian is able to understand the languages that intervention is provided.

**Results:**

Pilot study with feasibility assessment finished at early 2021. Recruitment of the wider RCT study is currently ongoing. The results from the pilot study and more detailed description about the intervention will be presented.

**Conclusions:**

This study with good national geographical coverage is a unique possibility to evaluate universal parenting program on promoting parenting behaviors associated with the promotion of optimal child emotional development. This study also provides population level information about parenting skills and child´s behavior.

**Disclosure:**

No significant relationships.

